# Shift of Volatile Organic Compounds (VOCs) in Gluten-Free Hemp-Enriched Sourdough Bread: A Metabolomic Approach

**DOI:** 10.3390/nu12041050

**Published:** 2020-04-10

**Authors:** Lorenzo Nissen, Alessandra Bordoni, Andrea Gianotti

**Affiliations:** 1 CIRI - Interdepartamental Centre of Agri-Food Industrial Research, Alma Mater Studiorum University of Bologna, P.za G. Goidanich 60, 47521 Cesena, FC, Italy; lorenzo.nissen@unibo.it (L.N.); andrea.gianotti@unibo.it (A.G.); 2 DiSTAL-Department of Agricultural and Food Sciences, Alma Mater Studiorum University of Bologna, Piazza Goidanich, 60–47521 Cesena (FC), Italy

**Keywords:** SPME-GC-MS, sourdough, hemp, flavoring compounds, bioactive compounds, multivariate analysis, gluten free

## Abstract

Hemp seed flour represents a potential ingredient for protein enrichment of gluten-free bakery products, the nutritional value of which could be further increased by fermentation with sourdough or with beneficial lactic acid bacteria strains. In this study, a metabolomic approach was used to evaluate the effect of hemp seed flour addition and sourdough fermentation on the production of flavoring and health-related volatile organic compounds (VOCs) in a gluten-free bread. Multivariate analysis of VOCs provided an in-depth description of the effects of hemp seed flour addition and sourdough fermentation on flavoring and bioactive compounds. In particular, an increased concentration of antimicrobial compounds, a larger spectrum of bioactive VOCs and a typical flavoring profile was evidenced in comparison to standard products. Furthermore, an increase of fermentation metabolites was observed in comparison to a standard dough, relating to abundances of 2-butanone-3-hydroxy, acetic acid, ethanol, and 1,4-butanediol. This study provides new insights on the evolution of flavoring and bioactive hemp seed flour constituents during sourdough fermentation, evidencing their retention in baked goods, and describes a new approach that could guide the formulation of innovative, fermented food with enhanced nutritional value.

## 1. Introduction

Celiac disease (CD) is a chronic systemic autoimmune disorder caused by a permanent intolerance to gluten proteins in genetically susceptible individuals. It affects 1% of the population in Europe [1], and its management requires exclusion of dietary gluten and the substitution of gluten-containing products with gluten-free (GF) products. The manufacturing of GF products is challenging not only from an organoleptic but also from a nutritional point of view. GF products are often nutritionally less adequate than standard products for the low protein and high fat, sugar, and salt content [2].

Hemp seed flour (HSF) represents a potential ingredient for GF bakery products. Beside its high protein content (33% *w*/*w*), HSF is characterized by a low content of saturated fats (4.5% *w*/*w*) and a good percentage of polyunsaturated fatty acids (PUFA) (38% *w*/*w*), including ω3 (8% *w*/*w*) and ω6 (27% *w*/*w*). Hemp seed flour is naturally free of cholesterol, it has a low glycemic index, 4% (*w*/*w*) of dietary fibers, and it is rich in magnesium (0.7% *w*/*w*) [3]. Furthermore, HSF contains around 300 mg/kg of polyphenols and a broad range of bioactive molecules such as flavonoids, terpenes, and cannabinoids [4] that have striking *in vitro* and *ex vivo* antioxidant activity [5]. Recently, 20% (*w*/*w*) HSF was used to enrich GF bread [6]. 

Fermentation with sourdough or with beneficial lactic acid bacteria (LAB) strains could further increase the nutritional value of HSF-enriched GF products since it provides direct (ingestion of beneficial bacteria) and indirect (ingestion of microbial metabolites) health benefits to consumers [7]. During fermentation, volatile organic compounds (VOCs) are synthesized naturally by microorganisms (as secondary metabolites) interacting with the food matrix. VOCs are organic molecules that include esters, alcohols, aldehydes, ketones, phenols, organic acids, terpenes, etc. Beyond their flavoring properties, various reports have shown the potential role of VOCs in human health, including their antioxidant, anti-inflammatory, anti-cancer, and anti-obesity activities [8,9]. Metabolomics is a promising tool to assess the safety, traceability, and quality of foods. In fermented foods, the metabolite profiling was applied to observe metabolite modifications during fermentation and to predict the sensorial and nutritional quality in different food matrices including dairy [10], bakery [11], tomato [12], and traditional fermented foods [13]. 

In this study, a metabolomic approach was used to evaluate VOCs production in a sourdough-fermented, GF bread enriched with HSF and to relate it to flavoring and health properties of the final product. 

## 2. Materials and Methods

### 2.1. Microbial Strains and Culture Conditions

Microbial strains belonged to the microbial collection of the Department of Agricultural and Food Sciences, University of Bologna (Italy) [11,14,15]. *Lactobacillus plantarum* 98a, *Lb. sanfrancisciensis* Bb12 and *Saccharomyces cerevisiae* LBS were obtained from 30% (*v*/*v*) glycerol stocks stored at -80 °C. Bacteria were propagated in de Man–Rogosa–Sharpe (MRS) (Oxoid, Thermo Scientific, Waltham, MA, USA) broth at 37 °C for at least 48 h and yeasts in Sabouraud broth (Oxoid, Thermo Scientific, USA) at 30 °C for 24 h. 

### 2.2. Doughs and Bread Preparation 

Flours were commercial organic certified products (Table S1). Experimental doughs (approximately 700 g) were prepared according to the formulation reported in Table S2. Maize and rice were partially substituted with 5.3 % (*w*/*w*) HSF to obtain a GF formulation suitable to be considered as a protein source. The list of samples and their codes are described in Table 1. Two types of doughs formulations were used: a standard type (S) including maize and rice flours and an HSF type (H) where HSF (5.3% *w*/*w*) replaced standard flours. The percentage of HSF to be used for enrichment was set based on the sensory characteristics of the final products. Both types were used for direct fermentations and for sourdough fermentations. Direct fermentations were as follows: (i) not inoculated (X); (ii) inoculated with Log_10_ 6 CFU/mL of an equal LAB mix of *L. sanfrancisciensis* Bb12 and *L. plantarum* 98a (L); (iii) inoculated with Log_10_ 7 CFU/mL of *S. cerevisiae* LBS (Y), and it was conducted for 18 h at 31 °C. Additionally, a direct fermentation with Y was conducted for 6 h at 31 °C. The sourdough fermentations were made by replacing 20% of H and S dough formulations with 140 g of direct L fermented doughs and inoculated with Log_10_ 7 CFU/mL of Y. The sourdoughs were then fermented for 6 h at 31 °C. All fermented doughs were baked at 180 °C for 20 min to produce breads (B). All samples were produced in triplicates in two independent experiments. 

### 2.3. Microbial Quantification during the Process

Microbial quantification was obtained by both culture-dependent and culture-independent protocols. The culture-dependent quantification was done by plating serial dilutions of the samples in physiological solution (0.9% NaCl). LAB were plated on MRS (de Man–Rogosa–Sharpe) (Oxoid, Thermo Fisher Scientific, Waltham, MA, USA) agar and cycloheximide (0.1 g/L) (Sigma, Saint Louis, MO, USA) and incubated aerobically for 24 h at 37 °C. Yeasts were plated on Sabouraud dextrose agar (Oxoid, Thermo Fisher Scientific, USA) and chloramphenicol (0.4 g/L) (Sigma, Saint Louis, MO, USA) and incubated aerobically for 48 h at 30 °C. Quantification was calculated as Log_10_ CFU/mL (Colony Forming Units/mL). Culture independent protocol was performed by qPCR with the SYBR Green I chemistry, applying genus specific primers as Lac1 for *Lactobacillus* spp., then named LAB, (forward:5’-GCAGCAGTAGGGAATCTTCCA-3’ and reverse: 5’-GCATTYCACCGCTACACATG-3’) [16] and ITS 23S for *S. cerevisiae* LBS, then named yeasts, (forward: 5’-GTTTCCGTAGGTGAACCTGC-3’and reverse: 5’-ATATGCTTAAGTTCAGCGGGT-3’) [17]. Extraction of bacterial DNA was obtained with Nucleo Spin Food DNA Extraction Kit (Macherey Naegel, Duren, Germany) prior a pre-treatment of 10 min at 20 Hz of ultra-pure water diluted doughs in a sonication bath. Genetic standards were prepared from relative PCR amplicons from pure cultures of the target bacterial species as described previously [18]. For both the targets, qPCR reaction on a RotorGene 6000 (Qiagen, Hilden, Germany) was set as previously described [18]. Sample reactions were conducted in triplicate, with positive, negative, and background control. Quantification was calculated as GCN/mL (Gene Copy Number/mL) and the value divided by three (the presumptive copies of a ribosome per cells), expressed as Log_10_ cells/mL [18,19]. 

### 2.4. pH Changes during the Process

The pH was determined at 20 °C with a pH meter (Crison, Alella, Spain) appropriately calibrated with three standard buffer solutions at pH 9.21, pH 4.00, and pH 2.00. The pH values were measured in duplicate at three different times to monitor the fermentation (Table S3).

### 2.5. Solid-Phase Microextraction-Gas Chromatography-Mass Spectrometry (SPME-GC-MS) 

Evaluation of volatile organic compounds (VOCs) was carried out on an Agilent 7890A Gas Chromatograph (Agilent Technologies, Santa Clara, CA, USA) coupled to an Agilent Technologies 5975 mass spectrometer operating in the electron impact mode (ionization voltage of 70 eV), equipped with a Chrompack CP-Wax 52 CB capillary column (50 m length, 0.32 mm ID) (Chrompack, Middelburg, The Netherlands). The SPME-GC-MS (solid phase micro-extraction gas chromatography–mass spectrometry) protocol and the identification of volatile compounds were done according to previous reports, with minor modifications [11,15]. Briefly, before each head space sampling, the fiber was exposed to the GC inlet for 10 min for thermal desorption at 250 °C in a blank sample. The samples were then equilibrated for 10 min at 40 °C. The SPME fiber was exposed to each sample for 40 min and finally the fiber was inserted into the injection port of the GC for a 10 min sample desorption. The temperature program was: 50 °C for 0 min, then programmed at 1.5 °C/min to 65 °C, and finally at 3.5 °C/min to 220 °C, which was maintained for 20 min. Injector, interface, and ion source temperatures were 250, 250, and 230 °C, respectively. Injections were carried out in splitless mode, and helium (3 mL/min) was used as carrier gas. Identification of molecules was carried out by comparing their retention times with those of pure compounds (Sigma, USA) and confirmed by searching mass spectra in the available databases (NIST version 2005 and Wiley version 1996) and literature [11,15,18,19]. Ethyl alcohol, 1,4-butandiol, acetic acid, and 2-butanone-3-hydroxy were absolutely quantified in mg/kg, while all other VOCs were relatively quantified in percentage.

### 2.6. Statistical Analyses 

All statistical analyses were performed using TIBCO Statistica 8.0 (Tibco Inc., Palo Alto, CA, USA). Normality was checked with the Shapiro–Wilk test, while homoscedasticity was evaluated with the Levene’s test [20]. Differences between all samples were evaluated with untargeted Analysis of Variance (ANOVA) set at *p* < 0.05. Multivariate analysis was conducted with principal component analysis (PCA), K-mean clustering, and MANOVA (*p* < 0.01). Pearson correlations were used to generate the heatmap of VOCs prior fermentation. For post hoc test, an HSD Tukey’s test was employed (*p* < 0.05). Except for the quantification in mg/kg of major metabolites, independently normalized data set was proposed for each chemical class of molecules. The data were normalized using the mean centering method [18,19]. All results are expressed as mean values obtained at least from duplicate batches in two independent experiments. 

## 3. Results

### 3.1. Microbial Quantification and pH Values during the Process

Quantification of LAB and yeasts obtained by plate count and qPCR are shown in Table 2. Results expressed as Log_10_ cell/g and represent the mean value of the two methods since they give similar results when are applied to quantify the evolution of a known and standardize inoculum in an essential food prototype made in a research laboratory [18,19]. In HSF sourdoughs, the microbial load resulted higher than in standard samples (*p* < 0.05). In all conditions, direct inoculation of LAB was the starter that mostly reduced pH during fermentation (Table S3). In particular, the strongest acidification was achieved in standard doughs after 18 h (SL18), attaining pH 3.60 ± 0.08. After 24 h of sourdough fermentation, the most acidified dough was the HSF-enriched one (YH+6), accounting for pH 4.11 ± 0.11. Acidification was faster in HSF doughs even after 6 h of direct fermentation with *S. cerevisiae* LBS (pH 5.81 ± 0.11).

### 3.2. Analysis of the Volatilome

Volatilome analysis identified more than 200 molecules and relatively quantified approximately 140. For a landscape description of the volatilome, two datasets normalized with the mean centering method were proposed: (i) one including not fermented cases and 126 significant molecules (*p* < 0.05) (Section 3.2.1) and (ii) one including all experimental cases and the sums of relative abundances of molecules grouped by chemical class, employed to compare the not fermented cases to the means of fermented cases (Section 3.2.2). Afterward, for a more specific investigation and to generate robust data trainings for multivariate analysis, two other options were chosen: (iii) the most abundant compounds (ethyl alcohol, acetic acid, 2-butanone-3-hydroxy, and 1,4-butandiol) were set apart and independently quantified in mg/kg using an internal standard as described previously [18,19] (Section 3.2.3); (iv) all other compounds for multivariate analyses (PCA, K-Means, and MANOVA) were obtained from five super-normalized data sets organized by different VOCs chemical class (Section 3.2.4). 

#### 3.2.1. Quantification of VOCs before fermentation

One-hundred-twenty-six VOCs, including 34 alcohols, 23 aldehydes, 29 ketones, 22 organic acids, and 18 alkenes, were detected in flours and in not fermented doughs. The data set was clustered by Pearson analysis in two major groups, the first including HSF samples and HSF not fermented doughs and the second including standard flour samples and related not fermented doughs. A higher quantity and a wider speciation of compounds was identified in HSF samples (HSF: 57 VOCs vs. 49 VOCs in standard flours; HSF doughs: 107 VOCs vs. 69 VOCs in standard doughs). In most cases, hexanal was one of the most abundant compounds, reaching the top level in HSF and HSF doughs. In these samples, octanone and ethylcyclopentanone were the second most abundant compounds. Of note, these ketones were not identified in standard samples. As well, 1-octen-3-ol and caryophyllene oxide were detected in significant amount only in HSF samples. The heatmap of relative quantification of VOCs in the different samples is reported in Figure S1.

#### 3.2.2. Effect of Fermentation

**Figure 1:**
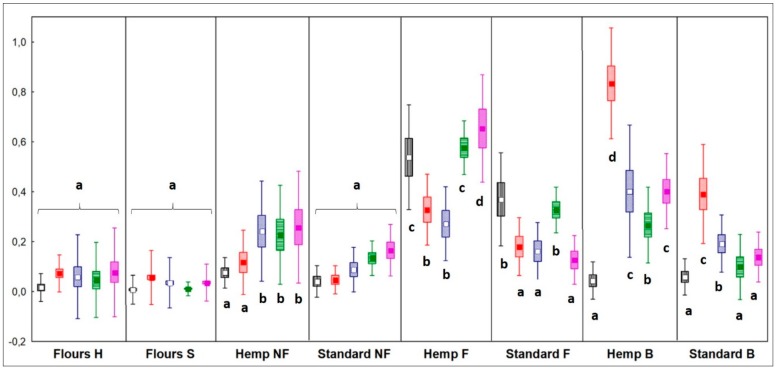
Relative quantification of total volatile organic compounds (VOCs) divided by chemical classes. Different letters indicate different significance values by Tukey’s HSD test (*p* < 0.05). Sample abbreviations: H = hemp seed enriched; S = standard; NF = not fermented; F = fermented; B = breads. Box = mean value; Rectangles = mean ± Standard Deviation (SD); Whiskers = mean + 1.96*SD. Black plots = alcohols; red plots = aldehydes; blue plots = ketones; green plots = organic acids; fuchsia plot = alkenes.

In HSF samples, fermentation caused a significant increase of VOCs in all chemical classes, while in standard samples only alcohols and organic acids significantly increased (*p* < 0.05). After baking, in HSF bread, the concentration of aldehydes, ketones, and organic acids was double compared to the standard breads, and that of alkenes was triple (Figure 1). 

#### 3.2.3. Quantifications of the Main Fermentation Metabolites

Quantification of main fermentation metabolites (mg/kg of fermented matrix) is reported in Table 3. Ethanol produced in yeast fermented HSF dough (HY18) scored higher than its control (SY18), while an opposite trend was observed when the fermentation process was performed by sourdough. Acetic acid in HSF-enriched doughs scored the highest concentration when directly fermented with LAB (LAB) for 18 h (HL18) accounting for 1.88-folds more than the relative control (SL18) (*p* < 0.05). The HSF-enriched sourdoughs (YH+6) produced 1.44-fold more acetic acid than the control, while in the baked samples, those directly fermented by LAB (HLB) had 3.1-fold more acetic acid than the control breads (SLB) (*p* < 0.05). In comparison to the control, HSF fermented doughs produced a similar content (p>0.05) of 1,4-butanediol, but in the HSF sourdough breads (YH+B) this compound was retained 1.6-folds more (*p* < 0.05) than in standard bread. *S. cerevisiae* LBS direct fermentation almost doubled 2-butanone-3-hydroxy concentration in HSF samples (HY18) and breads (HYB) compared to their controls.

#### 3.2.4. Multivariate Analysis of VOCs Organized by Different Chemical Classes

##### Alcohols

**Figure 2:**
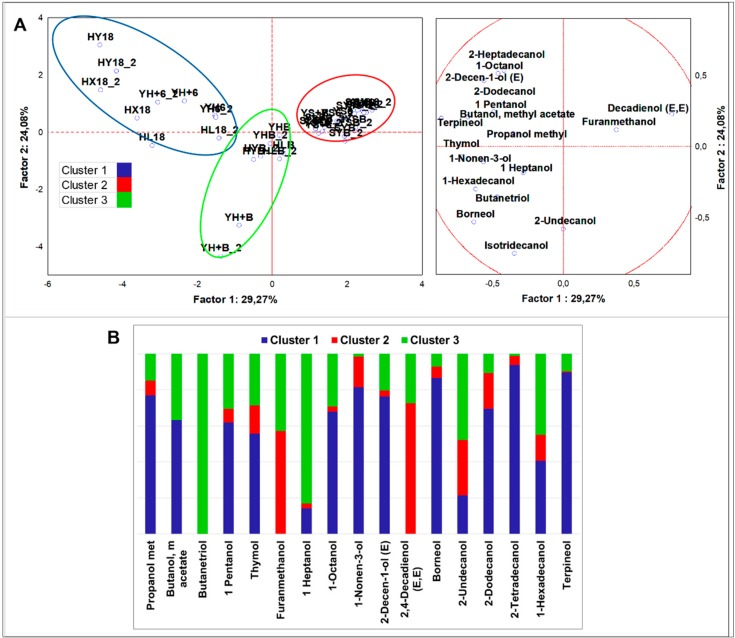
(**A**) Principle component analysis (PCA) of cases and variables on alcohols (*p* < 0.05); (**B**) K-means clustering analysis (at least *p* < 0.05).

To better evidence differences in the alcohol class, normalization of the dataset and statistical analysis were performed after exclusion of the most abundant compounds, ethanol and 1,4-butandiol. Moreover, flours and not fermented doughs were not included (n = 28) in the dataset due to the scarce abundance of alcohols. PCA grouped samples in three clusters (Figure 2). HSF samples were grouped in two clusters. Cluster 1 (all HSF doughs regardless fermentation) was described by higher concentration of almost every alcohol and by a typical signature made by thymol, borneol, and terpineol. Cluster 3 (all HSF breads) was described by top amounts of 1-heptanol, 1-nonen-3-ol, and 1,2,4-butanetriol, with the former as a distinct sign. The speciation of alcohols was comparable to HSF doughs, but alcohol concentrations were strongly decreased. Indigenous fermentation significantly fostered the production of butanol, methyl acetate, 1-nonanol, and hexadecanol, while sourdough fermentation promoted that of 1-octen-3-ol, octanol, 2-butyl, and 2-undecanol (Figure S2A,B). 

Direct fermentation performed by *S. cerevisiae* LBS was marked by higher cyclohexanol abundance, while LAB fermentation generated more 1,2,4-butanetriol. Thymol, borneol, and terpineol were robust descriptors of HSF enriched goods. Lastly, furanmethanol resulted 83% lower in the HSF fermented doughs and in the HSF breads in comparison to the control samples.

##### Aldehydes

**Figure 3:**
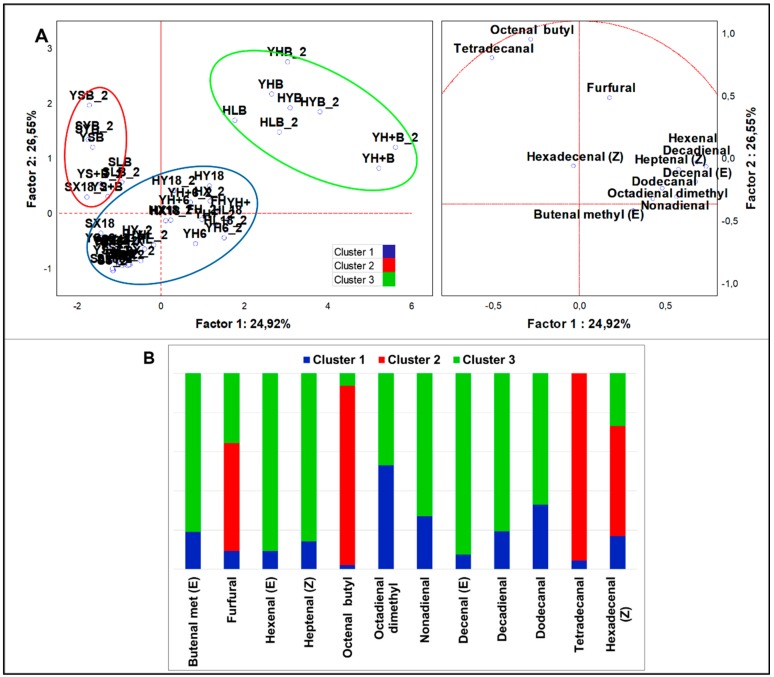
(**A**) PCA of cases and variables on aldehydes (*p* < 0.05); (**B**) K-means clustering analysis (at least *p* < 0.05).

From analysis of variance of all samples (n = 58), 10 aldehydes resulted significantly different (*p* < 0.05). PCA loadings (Figure 3A) were clustered in three sets by K-means analysis (Figure 3B). HSF samples were grouped in two clusters. Cluster 1 was the largest set (n = 41), including standard and HSF doughs prior to and after the fermentation. This cluster was described by high concentration just of 2,4-nonadienal (E,E), which accounted for about 50% of total aldehydes. Cluster 3 included all HSF breads and was characterized by a large speciation of aldehydes and by top abundance of 2-hexenal (E), 2-decenal (E), 2-heptenal (Z), 2-methyl-2-butenal, and 2,4-decadienal (E,E) compared to other clusters. In comparison to the cluster grouping standard breads, cluster 3 had a unique signature made of the aforementioned first three compounds. Results from MANOVA (*p* < 0.01) (Figure S2C,D) indicated that samples obtained by indigenous fermentation (HX18) had a major quantity of acetaldehyde and furfural, while samples made with sourdough had more 2-hexenal (E) and 2-octenal (Z). Samples directly fermented by *S. cerevisiae* LBS generated typical aldehydes, such as octadienal dimethyl (Z) and tetradecanal, while those fermented by LAB were characterized by 2,4-nonadienal (E,E). Interestingly, this analysis evidenced that HSF breads accounted for the top proportion in production of 2-heptenal (Z), octadienal dimethyl (Z), 2,4-decadienal (E,E), and dodecanal. Since these compounds were not found in the raw material it is conceivable that they were a result of bread making. On the contrary, 2-decenal (E) diminished drastically with bread making. 

##### Ketones

**Figure 4:**
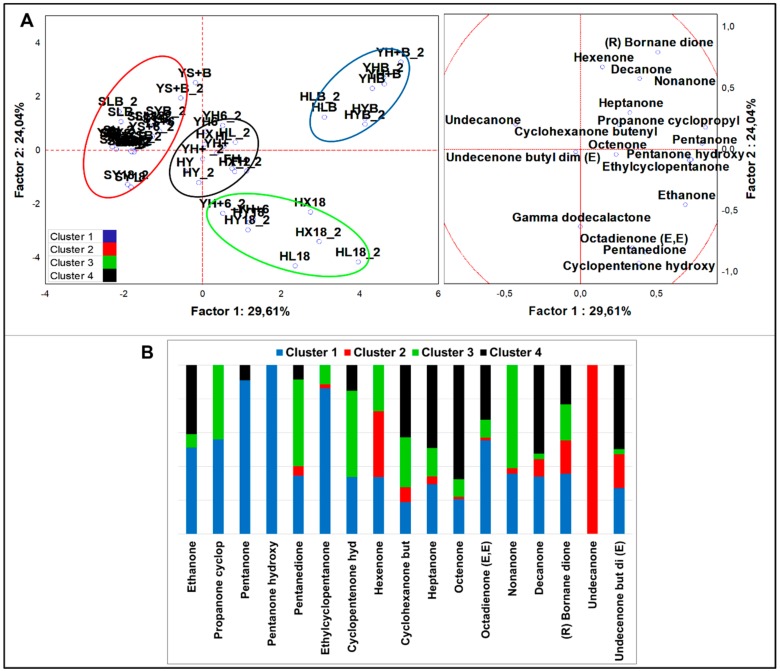
(**A**) PCA of cases and variables on ketones (*p* < 0.05); (**B**) K-means clustering analysis (at least *p* < 0.05).

Analysis of variance including all samples (n = 58) evidenced 18 significantly different ketones (*p* < 0.05). PCA loadings (Figure 4A) were clustered in four sets by K-means analysis (Figure 4B). HSF samples were contained in three clusters. Cluster 1 comprised all the HSF breads regardless of the type of fermentation. It was described by all ketones except 2-undecanone, with top concentration of ethylcyclopentanone, 2-pentanone-3-hydroxy, 2,4-octandienone (E,E), 2-pentanone, and (R)-bornane dione. Ethylcyclopentanone was the most abundant ketone among all variables and was quantified 7.52-folds more than in the runner-up cluster. Along with 2-pentanone and 2-pentanone-3-hydroxy, it represented the unique signature of HSF breads. All fermented HSF doughs except YH6 were grouped in cluster 3, which was described by 2-nonanone, 2,3-pentanedione, 2-cyclopentenone-3-hydroxy, and 2-propanone cyclopropyl. Lastly, cluster 4 comprised ten cases related to HSF not fermented doughs and was described by 12 different ketones, oct-3-en-2-one and (E)-6,10-dimethyl-undec-3-en-2-one being the best markers. From MANOVA (*p* < 0.01) (Figure S2E,F) emerged that the indigenous fermentation fostered the production of 2-pentanone-3-hydroxy and 2-pentadecanone, the sourdough that of 2-butanone and 2,10-undecadione 6-6-dimethyl (E), while direct fermentation was marked by higher 2-haxanone and hex-3-en-2-one abundances by *S. cerevisiae* LBS and LAB, respectively. In HSF-enriched products, concentration of significant ketones was 7.2-times higher than in standard samples. In addition, HSF products were described by ten ketones compared to eight, 2-cyclopentenone-3-hydroxy and (R)-bornane dione being evidenced only in HSF fermented doughs and breads.

##### Alkenes

**Figure 5:**
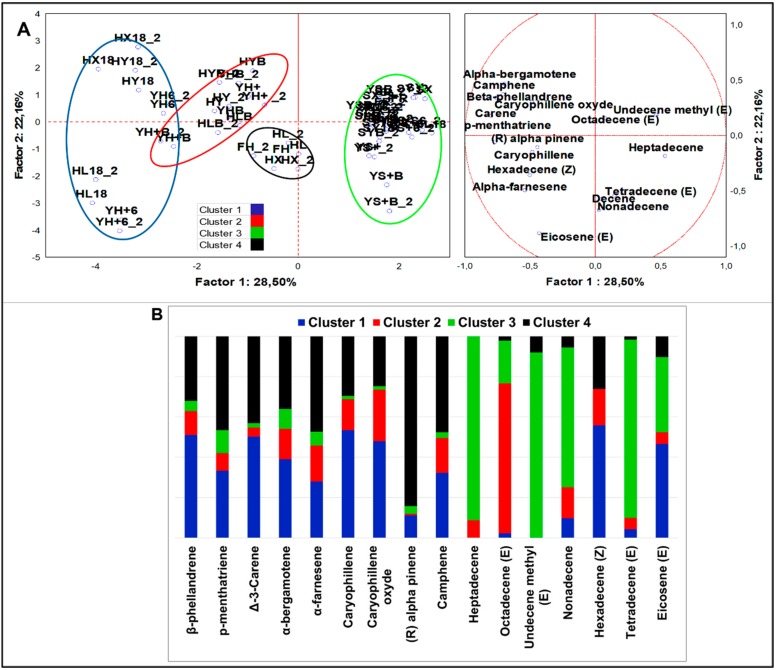
(**A**) PCA of cases and variables on alkenes (*p* < 0.05); (**B**) K-means clustering analysis (at least *p* < 0.05).

From analysis of variance including all samples (n = 58), 16 alkenes, notably, 9 terpenes and 7 α-olefins resulted significantly different (*p* < 0.05). PCA loadings (Figure 5A) were clustered in four sets by K-means analysis (Figure 5B). HSF samples were contained in three clusters, mainly described by terpenes, while standard samples were clustered separately and mainly described by α-olefins. Cluster 1 and 2 included HSF fermented doughs and HSF breads, respectively. HSF fermented doughs were described by nine terpenes and two α-olefins, with top abundance of β-phellandrene, ∆-3-carene, caryophillene oxide, and eicosene (E). ∆-3-carene was the most abundant alkene among all variables of the dataset. The HSF breads were described by a similar compound speciation but with a slightly minor abundance; in particular, ∆-3-carene and (R)-α-pinene were found in traces. Octadecene (Z) was the most abundant α-olefin in all HSF samples. Cluster 4 grouped only hemp seed flour and four not fermented HSF samples. It was described by all nine terpenes abundantly quantified and by traces of three α-olefins and minor load of hexadecene (Z). The most abundant terpenes were in order ∆-3-carene, (R)-α-pinene, camphene, and α-farnesene. Moreover, 84.3% of total (R)-α-pinene and more than 47.7% of total camphene were members of this cluster. From MANOVA (p < 0.01) (Figure S2G,H), it emerged that *S. cerevisiae* LBS generated larger production of α-bergamotene, while LAB fermentation induced a higher concentration of β-phellandrene and ∆-3-carene. Sourdough was the starter most influencing the alkene abundances; indeed, α-farnesene and four out of six α-olefins, including hexadecene (Z) and eicosene (E), were augmented by this type of fermentation. Independently from fermentation, β-phellandrene, ∆-3-carene, and caryophyllene were robust descriptors of HSF-enriched doughs or breads.

##### Organic Acids

**Figure 6:**
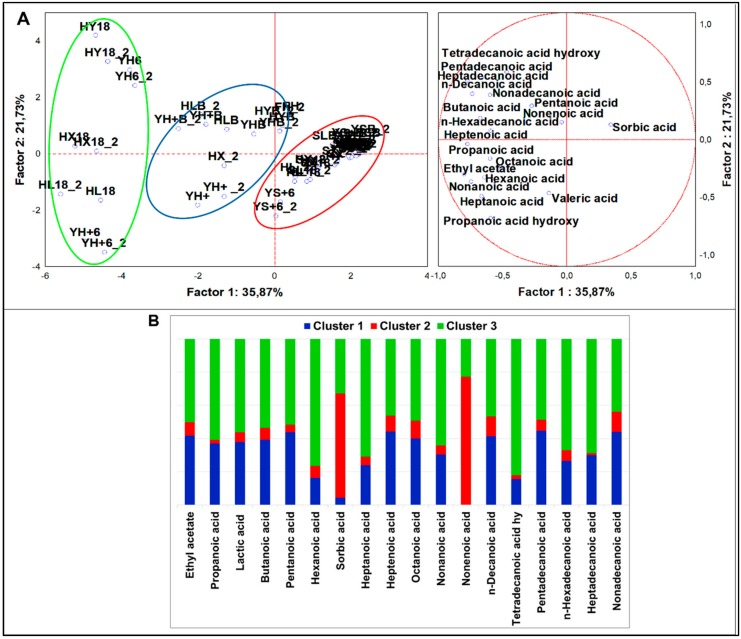
(**A**) PCA of cases and variables on organic acids (*p* < 0.05); (**B**) K-means clustering analysis (*p* < 0.05).

From analysis of variance including all samples (n = 58), 18 organic acids (4 short chain, 9 medium chain, and 5 long chain) resulted significantly different (*p* < 0.05). PCA loadings (Figure 6A) were clustered in four sets by K-means analysis (Figure 6B). HSF samples were grouped in two clusters. Cluster 1 consisted of several HSF samples, from the flour to the breads. It was described by a large speciation of organic acids contributing to a profile made of 17 different compounds, otherwise not plentifully quantified. The main VOCs were: pentadecanoic, n-decanoic, nonadecanoic, heptanoic, and heptadecanoic acids. Cluster 3 comprised most of the fermented HSF samples and was described by a generally higher abundance of organic acids but with a remarkably higher quantity of hexanoic, propanoic, lactic, tetradecanoic, n-hexadecanoic, octanoic, and heptanoic acids. Likely, in cluster 1, samples directly fermented with *S. cerevisiae* LBS were up in quadrant II, while those directly fermented with LAB and the sourdough were down in quadrant III. This latter distinction emerged when MANOVA was performed (*p* < 0.01) (Figure S2I,J). For example, LAB fostered the production of propanoic acid, accounting for 46.4% of the total, while the sourdough fostered that of lactic (51.5%), hexanoic (43.5%), heptanoic (43.4%), and octanoic (55%) acids. The indigenous flora pushed the production of nonanoic acid (50.1%), while *S. cerevisiae* LBS pushed that of pentadecanoic acid (49%). Interestingly, 32.84% of n-hexadecanoic acid was not derived from fermentation. In fact, its abundance was related to the 33.9% and 63.9% in HSF doughs and HSF flour, respectively. Besides, while medium-chain organic acids, such as heptanoic and nonanoic acids, were mainly present in HSF goods (91.1% and 92.7%, respectively), the long-chain organic acids, such as heptadecanoic and nonadecanoic acids, were majorly derived from FH, accounting for 62.7% and 60.2% of total values. 

## 4. Discussion

### 4.1. Microbial Growth and Major Fermentation Metabolites

In all HSF fermented samples, microbial growth was massive and higher than in the standard samples. Microbial growth after sourdough fermentation was higher than in the recent study by Nionelli et al., [21], probably due to the high adaptation ability of the strains we used as they were isolated from a sourdough ecosystem (wheat sourdough). The optimal performance of the strains was confirmed by the better acidification observed in this study than in the previous study [21]. An efficient dough acidification is an important characteristic of fermentation, since it inhibits spoilage microbes and increases bread loaf homogeneity and flavor. Moreover, HSF addition to the dough directly fermented with *S. cereviase* LBS provided the maximum production of ethanol, the most important descriptor for an efficient leavening process. The lower acetic acid loads observed in our study compared to the previous one [21] were probably due to the shorter fermentation time and the use of GF flours, which are generally considered less fermentable than wheat. Although the content of 2-butanone-3-hydroxy, which is essential for the structure and a pleasant aroma of the bakery product [22] decreased in HSF bread due to baking loss, it was still higher than in standard breads.

### 4.2. Multivariate Analysis of VOCs Sorted by Chemical Class

Flavoring characteristics and putative bioactivity of the main VOCs in HSF-enriched GF doughs and breads are summarized in Table 4.

#### 4.2.1. Alcohols

Among minor alcohols, 1-heptanol, 1-octen-3-ol, 1-nonen-3-ol, tetradecanol, borneol, thymol, and terpineol better discriminated HSF containing samples. 1-heptanol, 1-nonen-3-ol, and 1-octen-3-ol are associated to hemp drinks fermented with probiotics [18]. 1-heptanol is used as a flavoring agent conferring a typical olfactory issue described as musty, pungent, leafy, green [23]. 1-octen-3-ol derives from linoleic acid during oxidative breakdown, and it has antimicrobial activity against spoilage and opportunistic microbes [24]. Borneol, thymol, and terpineol are considered bioactive molecules and are reported to modulate beneficially the gut microbiome [25,28,32] and to possess anti-inflammatory and antioxidant activity [26,29,30,33]. Their exclusive presence in HSF products can be considered an added value, particularly because they were retained in HSF breads, with borneol as the most resistant (43% retained). The presence of theses alcohols could also have positive effects on shelf life due to their ability to contrast spoilage microorganisms [63,64,65]; of note, HSF samples evidenced the lowest concentration of 2-furanmethanol, which is considered a toxic compound [66,67]. A reduction of its content was recently observed also in hemp/wheat flour breads as the percent content of HSF increased [68].

#### 4.2.2. Aldehydes

HSF breads accounted for about 20% less furfural than standard breads. Furfural has a leading role in the aroma of bakery products [23], but it was recently investigated as a potential carcinogen [69,70]. The concentration of octadienal dimethyl (Z) naturally occurring in raw hemp seeds, significantly increased in HSF bread as effect of *S. cerevisiae* fermentation. Octadienal dimethyl (Z) is characterized by a nice aroma of lemon [35]. It has antimicrobial activity, mainly directed to food-borne and spoilage fungi [36,37,38], anti-inflammatory activity in rat experimental infection with pathogenic *Staphylococcus aureus* [39], and anti-hyperalgesic effects in combination with β-cyclodextrins in animal models [40]. HSF breads were also characterized by 2-heptenal (Z), which has a pleasant almond flavor and is recognized as an anti-inflammatory and anti-oxidant compound [41,42,43]. Our data evidenced a positive correlation of 2-heptenal (Z) with LAB growth in hemp seed substrate similar to fermented hemp drinks [18] and sourdough-fermented breads [23],

#### 4.2.3. Alkenes

β-phellandrene, ∆-3-carene, α-farnesene, camphene, and β-caryophyllene increased in HSF fermented dough and breads compared to HSF doughs prior fermentation. This effect, already observed in hemp drinks [18,19], was more evident after LAB fermentation. Besides, just smaller amounts of camphene and α-farnesene were quantified in standard samples. Due to the positive effects of this compounds (Table 4), their increase after fermentation deserves consideration.

Among the α-olefins, eicosene (E) was found in higher concentration in HSF than standard samples. Eicosene (E) is a part constituting ceramide, known as C20-sphingosine [54], and the increase of its dietary intake could promote an efficient sphingolipid metabolism. 

#### 4.2.4. Ketones

HSF samples were described by a larger speciation of ketones than standard samples at all stages of the process. Among ketones, 2-pentanone-3-hydroxy (2P3H) deserves attention. 2P3H is biosynthesized by plants and bacteria, mainly by *Bacillus* species [22] and lactic acid bacteria [71]. Accordingly, in our study, the abundance of this compound was fostered mainly by fermentation with the indigenous flora, which is realistically inhabited by species of *Bacillus* and in minor part by direct fermentation with LAB. The odor of 2P3H is described as caramel-sweet, buttery, and hay-like [56]. Moreover, this compound may decrease the content of toxic furanones by glycosylation [72], resulting in a less/not harmful product for human consumption.

#### 4.2.5. Organic Acids

In our study, the profile of organic acids of HSF samples was quantitatively and qualitatively superior to the standard one. Principally, a great amount of organic acids was discovered in the fermented HSF sourdoughs. In agreement with previous study [3,18], the increased concentration of propanoic, lactic, and medium-chain organic acids depended mainly on the fermentation process, while the high concentration of long-chain organic acids was more related to the enrichment with HSF. Nonanoic acid may represent a marker compound of hemp seed product [18]. Propanoic and lactic acids are flavoring compounds but are also involved in the quality and safety of fermented food due to their antimicrobial activity [18,23,57]. Besides, propanoic acid fits the new definition of prebiotics [58]. Since hexanoic and octanoic acids possess unpleasant flavoring traits (rancid-like) [23,59], the modulation of their content should be necessary and deserves further studies.

#### 4.2.6. Overall Considerations

The addition of HSF and the use of different fermentation types gave rise to specific VOCs profile predicting the organoleptic characteristics of bread. In sourdough bread, pleasant almond-like and lemon-like notes could be related to HSF addition or *S. cerevisiae* fermentation, respectively. In addition, increased traits of herbal, fruity, and floral notes (minor alcohols) could be provided by HSF addition both in yeast- and LAB-fermented bread, and sour and buttery nuances due to propanoic acid could be predicted in sourdough bread. The organoleptic impact of the increased concentration of medium and short-chain FA is difficult to predict since it greatly depends on the relative FA content. Indeed, except for fatty and creamy background (nonanoic), an excess of rancid-like notes may be ascribed to their unbalance. 

The health potential of bioactive compounds delivered in the experimental breads is hardly predictable for different reasons. First, although SPME-GC-MS is a quali/quantitative method, bioactive absolute concentration must be handled very carefully in estimating bioactivity. At present, the effective dose of most of the detected bioactive has been evaluated in specific experimental conditions and cannot be generalized. Moreover, the food matrix delivering the bioactives still represents a key parameter both for analysis and bioaccessibility.

The enrichment with HSF was based on the sensory characteristics and allowed to increase the protein concentration in the final products, although not to the extent needed for the claim “source of protein”, being about 11% instead of the 12% of the total energy required by the EU regulation (Regulation (EC) No 1924/2006, lastly amended by Regulation (EU) No 1047/2012). Of note, we did not add any flavoring or masking agents to avoid interference in the analyses. In the future, their addition could allow to further increase the percentage of HSF maintaining good sensory characteristics.

## 5. Conclusions

In this work, HSF was used to formulate a GF bread enriched in proteins. The bread was quantitatively and qualitatively analyzed throughout the process to evaluate fermentation performances and volatilome composition. The metabolomic profiles of HSF-enriched GF breads were considered to investigate the potential of hemp flour as a vehicle for the addition of flavoring and bioactive compounds in bakery products. Multivariate analysis on VOCs provided a deeper description of the effects of HSF addition and sourdough fermentation process on flavoring and bioactive compounds, mainly evidencing an increased concentration of antimicrobial compounds, a larger spectrum of bioactive VOCs, and a typical flavoring profile. 

The herein reported study was not aimed to the demonstration of any biological activity of the HSF-enriched bread, but it provided new insights on the modulation of flavoring and bioactive constituents of HSF during sourdough fermentation and evidenced the retention or even increase of the most of them in baked goods. Our approach allowed linking specific VOCs to different fermentation types and could be useful to tune the formation of desirable compounds in the final products. Although further studies coupling volatilome analysis to sensorial assessment are needed to meet consumers’ acceptance, the evaluation of the shift of VOCs could represent a comprehensive, sensitive, and reliable method guiding the formulation of innovative food with enhanced nutritional value. 

## Figures and Tables

**Table 1:** Description of samples codes.

Sample	Description
FH	Hemp seed flour
FM	Maize flour
FR	Rice flour
HX	Hemp seed dough not inoculated (direct)
HL	Hemp seed dough LAB inoculated (direct)
HY	Hemp seed dough *S. cerevisiae* LBS inoculated (direct)
SX	Standard dough not inoculated (direct)
SL	Standard dough LAB inoculated (direct)
SY	Standard dough *S. cerevisiae* LBS inoculated (direct)
YH+	Hemp seed dough added with sourdough
YS+	Standard dough added with sourdough
HX18	HX fermented 18 h
HL18	HL fermented 18 h
HY18	HY fermented 18 h
SX18	SX fermented 18 h
SL18	SL fermented 18 h
SY18	SY fermented 18 h
YH+6	YH+ fermented 6 h
YS+6	YS+ fermented 6 h
YH6	YH * fermented 6 h
YS6	YS * fermented 6 h
YH+B	Bread from YH+6
YS+B	Bread from YS+6
YHB	Bread from YH6
YSB	Bread from YS6
HLB	Bread from HL18
HYB	Bread from HY18
SLB	Bread from SL18
SYB	Bread from SY18

* same formulations of HY and SY, respectively.

**Table 2:** Mean values of lactic acid bacteria (LAB) and yeast growth (Log_10_ cells/g).

Sample	LAB	*S. cerevisiae* LBS
SX	2.60 ± 0.04 ^a^	3.90 ± 0.05 ^b^
SL	7.02 ± 0.26 ^c^	2.30 ± 0.09 ^a^
SY	5.30 ± 0.06 ^b^	6.60 ± 0.07 ^c^
HX	3.78 ± 0.08 ^b^	4.48 ± 0.04 ^b^
HL	7.15 ± 0.25 ^c^	3.48 ± 0.05 ^a^
HY	3.30 ± 0.08 ^a^	6.60 ± 0.09 ^c^
SX18	6.32 ± 0.10 ^b^	5.90 ± 0.11 ^b^
SL18	9.58 ± 0.34 ^d^	5.00 ± 0.11 ^b^
SY18	6.45 ± 0.14 ^c^	7.84 ± 0.04 ^c^
HX18	7.31 ± 0.29 ^c^	6.23 ± 0.04 ^c^
HL18	9.74 ± 0.19 ^d^	6.90 ± 0.14 ^c^
HY18	6.60 ± 0.15 ^c^	8.72 ± 0.16 ^d^
YS+	9.86 ± 0.24 ^d^	6.92 ± 0.07 ^c^
YH+	9.81 ± 0.21 ^d^	6.99 ± 0.13 ^c^
YS	5.71 ± 0.22 ^c^	6.33 ± 0.14 ^b^
YH	4.31 ± 0.15 ^b^	6.20 ± 0.07 ^b^
YS+6	10.97 ± 0.31 ^d^	7.27 ± 0.11 ^c^
YH+6	11.16 ± 0.23 ^d^	8.23 ± 0.19 ^c^
YS6	6.53 ± 0.19 ^c^	7.14 ± 0.14 ^c^
YH6	5.42 ± 0.09 ^b^	6.89 ± 0.14 ^c^

Different letters in the same column indicate statistical significance (at least *p* < 0.05). For samples abbreviations see Table 1.

**Table 3:** Quantification (mg/kg of fermented matrix) of the main fermentation metabolites.

Sample	Ethyl alcohol	Acetic acid	2-butanone-3-hydroxy	1,4-Butanediol
FR	tr. *	n.d.	n.d.	tr.
FM	n.d.^†^	n.d.	n.d.	n.d.
FH	n.d.	n.d.	n.d.	n.d.
HX	n.d.	n.d.	n.d.	n.d.
HL	tr.	0.34 ± 0.03 ^a^	n.d.	n.d.
HY	tr.	0.13 ± 0.05 ^a^	n.d.	n.d.
SX	tr.	n.d.	n.d.	n.d.
SL	tr.	n.d.	n.d.	tr.
SY	tr.	n.d.	n.d.	tr.
YH+	6.79 ± 1.06 ^b^	1.82 ± 0.82 ^a^	0.42 ± 0.17 ^a^	7.67 ± 1.02 ^c^
YS+	4.03 ± 0.72 ^b^	0.33 ± 0.11 ^a^	0.37 ± 0.09 ^a^	6.46 ± 1.32 ^b^
HX18	14.10 ± 0.79 ^c^	11.98 ± 0.68 ^c^	9.40 ± 0.69 ^c^	3.33 ± 0.56 ^b^
HL18	25.97 ± 0.69 ^c^	25.18 ± 2.26 ^d^	12.44 ± 1.69 ^c^	8.85 ± 1.58 ^c^
HY18	29.19 ± 3.00 ^c^	8.53 ± 1.83 ^c^	16.61 ± 1.99 ^c^	9.64 ± 1.23 ^c^
SX18	16.17 ± 2.08 ^c^	2.94 ± 0.07 ^b^	7.11 ± 2.02 ^b^	2.02 ± 0.34 ^a^
SL18	23.82 ± 1.54 ^c^	13.33 ± 1.57 ^c^	8.84 ± 0.99 ^c^	7.86 ± 0.49 ^c^
SY18	21.30 ± 2.65 ^c^	6.31 ± 1.06 ^b^	9.08 ± 0.85 ^c^	9.90 ± 1.37 ^c^
YH6	12.77 ± 1.90 ^c^	7.94 ± 0.42 ^c^	10.61 ± 1.44 ^c^	7.98 ± 1.28 ^c^
YS6	13.74 ± 2.32 ^c^	6.44 ± 0.55 ^b^	6.12 ± 0.55 ^b^	8.40 ± 1.19 ^c^
YH+6	15.03 ± 3.16 ^c^	18.60 ± 3.32 ^d^	10.14 ± 1.69 ^c^	23.75 ± 3.21 ^d^
YS+6	16.19 ± 2.13 ^c^	13.26 ± 2.41 ^d^	11.84 ± 1.05 ^c^	18.93 ± 2.03 ^d^
HLB	0.11 ± 0.04 ^a^	8.04 ± 1.07 ^c^	6.34 ± 0.18 ^b^	3.13 ± 0.64 ^b^
HYB	0.17 ± 0.02 ^a^	4.10 ± 0.11 ^b^	10.29 ± 1.54 ^c^	3.56 ± 1.04 ^b^
SLB	0.45 ± 0.09 ^a^	2.73 ± 0.66 ^b^	4.45 ± 0.44 ^b^	2.22 ± 0.43 ^a^
SYB	0.39 ± 0.08 ^a^	n.d.	6.77 ± 0.99 ^b^	2.95 ± 0.78 ^b^
YHB	0.63 ± 0.12 ^a^	0.45 ± 0.28 ^a^	6.76 ± 1.12 ^b^	1.95 ± 0.32 ^a^
YSB	0.14 ± 0.09 ^a^	tr.	5.45 ± 1.30 ^b^	1.45 ± 0.78 ^a^
YH+B	3.63 ± 0.98 ^b^	7.99 ± 1.51 ^c^	8.88 ± 0.87 ^c^	6.99 ± 1.21^c^
YS+B	2.87 ± 0.34 ^b^	5.33 ± 1.10 ^b^	6.87 ± 0.55 ^b^	4.33 ± 2.65 ^b^

Values are means of two replicates and two different batches. * traces = values < 0.1 mg/kg; † n.d. = not determined. Different letters in the same column indicate significant differences (at least *p* < 0.05). For samples abbreviations see Table 1.

**Table 4:** Flavoring and bioactivity of discriminant VOCs of hemp seed flour (HSF) fermented doughs and breads.

Compounds	Flavoring	Bioactivity	References
1-heptanol	musty, pungent, leafy green		[18,23]
1-octen-3-ol		antimicrobial activity against spoilage and opportunistic microbes	[24]
borneol	pine, wood, camphor	contrast spoilage microorganism bacterial foodborne and entero-pathogens; anti-inflammatory and antioxidant capacities for the treatment of ulcerative colitis; added to drinking water of rats for 7 days lowered the level of oxidative DNA lesions induced in their hepatocytes.	[23,25,26,27]
thymol	herbal, thyme, phenolic, medicinal, camphor	contrast spoilage microorganism bacterial foodborne and entero-pathogens; anti-inflammatory and antioxidant in human preadipocytes and in neuroprotection of rotenone-induced rat model of Parkinson’s disease.	[28,29,30,31]
terpineol	pine, terpene, lilac, citrus, woody, floral	contrast spoilage microorganism bacterial foodborne and entero-pathogens; anti-inflammatory and antioxidant in LPS-induced cell line.	[32,33,34]
octadienal dimethyl	nice aroma of lemon	counteract spoilage molds of breads; *in vitro* is reported to have antimicrobial potential to food borne and spoilage fungi; anti-inflammatory activity in experimental infection with pathogenic *Staphylococcus aureus;* anti-hyperalgesic effects in combination with β-cyclodextrins in animal models	[35,36,37,38,39,40]
2-heptenal (Z)	pleasant almond flavor	associated to different plant-based products with anti-inflammatory and anti-oxidant activities.	[23,41,42,43]
∆-3-carene	harsh, terpene-like, coniferous	active against spoilage microbes, food-borne pathogens, and pathogenic *E. coli.*	[31,44,45]
β-caryophillene oxide	dry, wood, cedarwood, carrot	anti-inflammatory and analgesic effects in different mouse models of inflammatory pain; antibacterial capacity versus *Helicobacter pylori.*	[31,46,47,48]
β-caryophillene	woody-spicy, dry and tenacious	known as “dietary cannabinoid”, it has been shown to be orally bioavailable; *C. sativa* essential oils bearing up to 13% of this compounds is effective against several opportunistic and spoilage microorganisms including *Helicobacter pylori*; prevents structural alteration of the myocardium; effective against LPS-induced oligodendrocyte toxicity; prevention of lipid accumulation and improvement of glucose uptake; insulinotropic and antidiabetic effects	[31,34,48,49,50,51,52,53]
Eicosene (E)		is a part constituting ceramide (Sphingosine); cardioprotective effects; on mouse model can be effective on treating metabolic disorder; in human plasma binds to high-density lipoprotein and exhibit anti-atherogenic properties	[45,54,55]
1-pentanone-3-hydroxy	caramel-sweet, buttery, and hay-like	is converted during glycosylation of toxic furanones	[56]
propanoic acid	typical sharp, acrid, vinegar, sour taste	Inhibition of ubiquitous bacilli, spoilage microbes and food-borne pathogens; prebiotics; fostering of the selective growth of probiotics in the gut; stimulation of epithelial immune function	[18,23,57,58]
lactic acid	sharp, acrid, vinegar, sour taste buttery nuance	inhibition of ubiquitous bacilli, spoilage microbes and food-borne pathogens	[18,23,57]
hexanoic acid	rancid-like	inhibition of molds in bread	[23,59]
heptanoic acid	rancid-like		[23]
octanoic acid	rancid-like	binding to -OH of serine residues of ghrelin activate the hormone and regulate hunger; in combination to antioxidant compounds produces esters lipophenols that have stronger and more stable host antioxidant activity;	[23,59,60,61]
nonanoic acid	fatty, waxy, and cheesy with a mild sweet creamy background	effective on excessive calorie burning, inducing weight loss	[18,23,59,62]

## Supplementary Materials

Supplementary materials can be found at https://www.mdpi.com/2072-6643/12/4/1050/s1. Figure S1: Heatmap of relative quantification of means of VOCs of flour samples and not fermented dough samples; Figure S2: MANOVA (p < 0.01) of VOCs on samples; Table S1: Proximate composition of flours (w/w); Table S2: Dough formulations; Table S3: pH values.

## References

[1] Allen B., Orfila C. (2018). The Availability and Nutritional Adequacy of Gluten-Free Bread and Pasta. Nutrients.

[2] Melini V., Melini F. (2019). Gluten-free diet: Gaps and needs for a healthier diet. Nutrients.

[3] Ross S.A., EI Sohly H.N., EI Kashoury E.A., El Sohly M.A. (1996). Fatty acids of Cannabis seeds. Phytochem. Anal..

[4] El Sohly M.A., Radwan M.M., Gul W., Chandra S., Galal A. (2017). Phytochemistry of *Cannabis sativa* L.. Prog. Chem. Org. Nat. Prod..

[5] Frassinetti S., Moccia E., Caltavuturo L., Gabriele M., Longo V., Bellani L., Giorgi G., Giorgetti L. (2018). Nutraceutical potential of hemp (*Cannabis sativa* L.) seeds and sprouts. Food Chem..

[6] Korus J., Witczak M., Ziobro R., Juszczak L. (2017). Hemp (*Cannabis sativa* subsp. *sativa*) flour and protein preparation as natural nutrients and structure forming agents in starch based gluten-free bread. LWT-Food Sci. Technol.

[7] Gobbetti M., Di Cagno R., de Angelis M. (2010). Functional microorganisms for functional food quality. Crit. Rev. Food Sci. Nutr..

[8] Ayseli M.T., Ipek Ayseli Y. (2016). Flavors of the future: Health benefits of flavor precursors and volatile compounds in plant foods. Trends Food Sci Technol..

[9] Mota-Gutierrez J., Barbosa-Pereira L., Ferrocino I., Cocolin L. (2019). Traceability of Functional Volatile Compounds Generated on Inoculated Cocoa Fermentation and Its Potential Health Benefits. Nutrients.

[10] Mozzi F., Ortiz M.E., Bleckwedel J., De Vuyst L., Pescuma M. (2013). Metabolomics as a tool for the comprehensive understanding of fermented and functional foods with lactic acid bacteria. Food Res. Int..

[11] Taneyo-Saa D.T., Nissen L., Gianotti A. (2019). Metabolomic approach to study the impact of flour type and fermentation process on volatile profile of bakery products. Food Res. Int..

[12] Kim E.J., Park S.E., Seo S.H., Kweon O.C., Son H.S. (2019). A GC–MS based metabolic profiling of fermented tomato by lactic acid bacteria. Appl. Biol. Chem..

[13] Rizo J., Guillén D., Farrés A., Díaz-Ruiz G., Sánchez S., Wacher C., Rodríguez-Sanoja R. (2018). Omics in traditional vegetable fermented foods and beverages. Crit. Rev. Food Sci. Nutr..

[14] Babini E., Tagliazucchi D., Martini S., Dei Più L., Gianotti A. (2017). LC-ESI-QTOF-MS identification of novel antioxidant peptides obtained by enzymatic and microbial hydrolysis of vegetable proteins. Food Chem..

[15] Taneyo-Saa D.T., Di Silvestro R., Nissen L., Dinelli G., Gianotti A. (2018). Effect of sourdough fermentation and baking process severity on bioactive fiber compounds in immature and ripe wheat flour bread. Food Sci. Technol..

[16] Castillo M., Martin-Orue S.M., Manzanilla E.G., Badiola I., Martin M., Gasa M.J. (2006). Quantification of total bacteria, enterobacteria and LAB populations in pig digesta by real-time PCR. Vet. Microbiol..

[17] Foschino R., Gallina S., Andrighetto C., Rossetti L., Galli A. (2004). Comparison of cultural methods for the identification and molecular investigation of yeasts from sourdoughs for Italian sweet baked products. FEMS Yeast Res..

[18] Nissen L., Demircan B., Taneyo-Saa D.L., Gianotti A. (2019). Shift of Aromatic Profile in Probiotic Hemp Drink Formulations: A Metabolomic Approach. Microorganisms.

[19] Nissen L., di Carlo E., Gianotti A. (2020). Prebiotic potential of hemp blended drinks fermented by probiotics. Food Research International. Food Res. Int..

[20] Granato D., de Araujo Calado M.V., Jarvis B. (2014). Observations on the use of statistical methods in food science and technology. Food Res. Int..

[21] Nionelli L., Montemurro M., Pontonio E., Vernia M., Gobbetti M., Rizzello C.G. (2018). Pro-technological and functional characterization of lactic acid bacteria to be used as starters for hemp (*Cannabis sativa* L.) sourdough fermentation and wheat bread fortification. Int. J. Food Microbiol..

[22] Xiao Z., Wang L., Gu R., Zhao J., Hou X., Zhu H. (2017). Hydroxy-pentanones production by *Bacillus* sp. H15-1 and its complete genome sequence. J. Biotechnol..

[23] Petel C., Prost C., Onno B. (2017). Sourdough volatile compounds and their contribution to bread: A review. Trends Food Sci. Technol..

[24] Xiong C., Li Q., Li S., Chen C., Chen Z., Huang W. (2017). In vitro antimicrobial activities and mechanism of 1-octen-3-ol against food-related bacteria and pathogenic fungi. J. Oleo Sci..

[25] Diniz-Silva H.T., Ramalho Brandão L., de Sousa Galvão M., Madruga M.S., Ferreira Maciela J., Leite de Souza E., Magnani M. (2020). Survival of Lactobacillus acidophilus LA-5 and Escherichia coli O157:H7 in Minas Frescal cheese made with oregano and rosemary essential oils. Food Microbiol..

[26] Stan M.S., Voicu S.N., Caruntu S., Nica I.C., Olah N.-K., Burtescu R., Balta C., Rosu M., Herman H., Hermenean A. (2019). Antioxidant and Anti-Inflammatory Properties of a *Thuja occidentalis* Mother Tincture for the Treatment of Ulcerative Colitis. Antioxidants.

[27] Horvathova E., Kozics K., Srancikova A., Hunakova L., Galova E., Sevcovicova A., Slamenova D. (2012). Borneol administration protects primary rat hepatocytes against exogenous oxidative DNA damage. Mutagenesis.

[28] Cusimano M.G., Di Stefano V., La Giglia M., Di Marco Lo Presti V., Schillaci D., Pomilio F., Vitale M. (2020). Control of Growth and Persistence of *Listeria monocytogenes* and β-Lactam-Resistant *Escherichia coli* by Thymol in Food Processing Settings. Molecules.

[29] Bordoni L., Fedeli D., Nasuti C., Maggi F., Papa F., Wabitsch M., De Caterina R., Gabbianelli R. (2019). Antioxidant and Anti-Inflammatory Properties of Nigella sativa Oil in Human Pre-Adipocytes. Antioxidants.

[30] Javed H., Azimullah S., Meeran M.N., Ansari S.A., Ojha S. (2019). Neuroprotective Effects of Thymol, a Dietary Monoterpene Against Dopaminergic Neurodegeneration in Rotenone-Induced Rat Model of Parkinson’s Disease. Int. J. Mol. Sci..

[31] The Good Scents Company Information System. http://www.thegoodscentscompany.com/.

[32] Korona-Glowniak I., Glowniak-Lipa A., Ludwiczuk A., Baj T., Malm A. (2020). The In Vitro Activity of Essential Oils against Helicobacter Pylori Growth and Urease Activity. Molecules.

[33] Li R., Yang J.-J., Song X.-Z., Wang Y.-F., Corlett R.T., Xu Y.-K., Hu H.-B. (2020). Chemical Composition and the Cytotoxic, Antimicrobial, and Anti-Inflammatory Activities of the Fruit Peel Essential Oil from *Spondias pinnata* (Anacardiaceae) in Xishuangbanna, Southwest China. Molecules.

[34] Askari V.R., Shafiee-Nick R. (2019). The protective effects of β-caryophyllene on LPS-induced primary microglia M1/M2 imbalance: A mechanistic evaluation. Life Sci..

[35] Habib S., Gupta P., Bhat S., Gupta J. (2020). In silico, in-vitro and in vivo screening of biological activities of citral. Int. J. Vitam. Nutr. Res..

[36] Garcia M.V., Copetti M.V. (2019). Alternative methods for mould spoilage control in bread and bakery products. Int. Food Res. J..

[37] Perczak A., Gwiazdowska D., Gwiazdowski R., Juś K., Marchwińska K., Waśkiewicz A. (2020). The Inhibitory Potential of Selected Essential Oils on Fusarium spp. Growth and Mycotoxins Biosynthesis in Maize Seeds. Pathogens.

[38] Ju J., Xie Y., Yu H., Guo Y., Cheng Y., Zhang R., Yao W. (2020). Synergistic inhibition effect of citral and eugenol against Aspergillus niger and their application in bread preservation. Food Chem..

[39] Martins H.B., Selis N.D., Souza C.L., Nascimento F.S., Pacheco de Carvalho S., D’Oliveira Gusmão L., dos Santos Nascimento J., Pereira Brito K., Itana de Souza S., Vasconcelos de Oliveira M. (2017). Anti-Inflammatory Activity of the Essential Oil Citral in Experimental Infection with *Staphylococcus aureus* in a Model Air Pouch. Evid. Based Complementary Altern. Med..

[40] Campos C.A., Lima B.S., Trindade G.G.G., Souza E.P.B.S.S., Mota D.S.A., Heimfarth L., Quintans J.S.S., Quintans-Júnior L.J., Sussuchi E.M., Sarmento V.H.V. (2019). Anti-hyperalgesic and anti-inflammatory effects of citral with β-cyclodextrin and hydroxypropyl-β-cyclodextrin inclusion complexes in animal models. Life Sci..

[41] Costa A.M.M., Silva L.O., Torres A.G. (2019). Chemical composition of commercial cold-pressed pomegranate (*Punica granatum*) seed oil from Turkey and Israel, and the use of bioactive compounds for samples’ origin preliminary discrimination. J. Food Compos. Anal..

[42] Tenuta M.C., Tundis R., Xiao J., Loizzo M.R., Dugay A., Deguin B. (2019). Arbutus species (Ericaceae) as source of valuable bioactive products. Crit. Rev. Food Sci. Nutr..

[43] Karabagias V.K., Karabagias I.K., Gatzias I., Badeka A.V. (2020). Prickly pear seed oil by shelf-grown cactus fruits: Waste or Maste?. Processes.

[44] Božik M., Cejnar P., Šašková M., Nový P., Maršík P., Klouček P. (2018). Stress response of Escherichia coli to essential oil components—Insights on low-molecular-weight proteins from MALDI-TOF. Sci. Rep..

[45] Hermier D., Lan A., Tellier F., Blais A., Grauso Culetto M., Mathé V., Bellec Y., Gissot L., Schmidely P., Faure J.D. (2020). Intestinal Availability and Metabolic Effects of Dietary Camelina Sphingolipids during the Metabolic Syndrome Onset in Mice. J. Agric. Food Chem..

[46] Alberti T.B., Barbosa W.L., Vieira J.L., Raposo N.R., Dutra R.C. (2017). (-)-β-Caryophyllene, a CB2 Receptor-Selective Phytocannabinoid, Suppresses Motor Paralysis and Neuroinflammation in a Murine Model of Multiple Sclerosis. Int. J. Mol. Sci..

[47] Aly E., Khajah M.A., Masocha W. (2020). β-Caryophyllene, a CB2-Receptor-Selective Phytocannabinoid, Suppresses Mechanical Allodynia in a Mouse Model of Antiretroviral-Induced Neuropathic Pain. Molecules.

[48] Woo H.J., Yang J.Y., Lee M.H., Kim H.W., Kwon H.J., Park M., Kim S.-K., Park S.-Y., Kim S.-H., Kim J.-B. (2020). Inhibitory Effects of β-Caryophyllene on Helicobacter pylori Infection In Vitro and In Vivo. Int. J. Mol. Sci..

[49] Gertsch J., Leonti M., Raduner S., Racz I., Chen J.Z., Xie X.Q., Altmann K.H., Karsak M., Zimmer A. (2008). Beta-caryophyllene is a dietary cannabinoid. Proc. Natl. Acad. Sci. USA.

[50] Geddo F., Scandiffio R., Antoniotti S., Cottone E., Querio G., Maffei M.E., Bovolin P., Gallo M.P. (2019). PipeNig®-FL, a Fluid Extract of Black Pepper (*Piper Nigrum* L.) with a High Standardized Content of *Trans-*β-Caryophyllene, Reduces Lipid Accumulation in 3T3-L1 Preadipocytes and Improves Glucose Uptake in C2C12 Myotubes. Nutrients.

[51] Kumawat V.S., Kaur G. (2020). Insulinotropic and antidiabetic effects of β-caryophyllene with l-arginine in type 2 diabetic rats. J. Food Biochem..

[52] Nissen L., Stefanini I., Grandi S., Sgorbati B., Biavati B., Monti A. (2010). Characterization and antimicrobial activity of essential oils of industrial hemp varieties (*Cannabis sativa* L.). Fitoterapia.

[53] Al-Taee H., Azimullah S., Nagoor Meeran M.F., Alaraj Almheiri M.K., Al Jasmi R.A., Tariq S., Khan M.A.B., Adeghate E., Ojha S. (2019). Beta-caryophyllene, a dietary phytocannabinoid attenuates oxidative stress, inflammation, apoptosis and prevents structural alterations of the myocardium against doxorubicin-induced acute cardiotoxicity in rats: An in vitro and in vivo study. Eur. J. Pharmacol..

[54] Chiricozzi E., Lunghi G., Di Biase E., Fazzari M., Sonnino S., Mauri L. (2020). GM1 Ganglioside Is A Key Factor in Maintaining the Mammalian Neuronal Functions Avoiding Neurodegeneration. Int. J. Mol. Sci..

[55] Millar C.L., Norris G.H., Vitols A., Garcia C., Seibel S., Anto L., Blesso C.N. (2019). Dietary Egg Sphingomyelin Prevents Aortic Root Plaque Accumulation in Apolipoprotein-E Knockout Mice. Nutrients.

[56] Neuser F., Zorn H., Berger R.G. (2000). Generation of Odorous Acyloins by Yeast Pyruvate Decarboxylases and Their Occurrence in Sherry and Soy Sauce. J. Agric. Food Chem..

[57] Bartkiene E., Schleining G., Krungleviciute V., Zadeike D., Zavistanaviciute P., Dimaite I., Kuzmaite I., Riskeviciene V., Joudeikine G. (2016). Development and quality evaluation of lacto-fermented product based on hulled and not hulled hempseed (*Cannabis sativa* L.). Food Sci. Technol..

[58] Gibson G.R., Hutkins R., Sanders M.E., Prescott S.L., Reimer R.A., Salminen S.J., Scott K., Stanton C., Swanson K.S., Cani P.D. (2017). Expert consensus document: The International Scientific Association for Probiotics and Prebiotics (ISAPP) consensus statement on the definition and scope of prebiotics. Nat. Rev. Gastroenterol. Hepatol..

[59] Martena B., Pfeuffer M., Schrezenmeir J. (2006). Medium-chain triglycerides. Int. Dairy J..

[60] Oh W.Y., Shahidi F. (2018). Antioxidant activity of resveratrol ester derivatives in food and biological model systems. Food Chem..

[61] St-Onge M.P., Jones P.J. (2002). Physiological effects of medium-chain triglycerides: Potential agents in the prevention of obesity. J. Nutr..

[62] Rego Costa A.C., Rosado E.L., Soares-Mota M. (2012). Influence of the dietary intake of medium chain triglycerides on body composition, energy expenditure and satiety: A systematic review. Nutr. Hosp..

[63] Ji H., Kim H., Beuchat L.R., Ryu J.H. (2019). Synergistic antimicrobial activities of essential oil vapours against *Penicillium corylophilum* on a laboratory medium and beef jerky. Int. J. Food Microbiol..

[64] Císarová M., Hleb L., Medo J., Tančinová D., Mašková Z., Čuboňc J., Kováčikd A., Foltinová D., Božike M., Klouček P. (2020). The in vitro and in situ effect of selected essential oils in vapour phase against bread spoilage toxicogenic aspergilli. Food Control.

[65] Matulyte I., Jekabsone A., Jankauskaite L., Zavistanaviciute P., Sakiene V., Bartkiene E., Ruzauskas M., Kopustinskiene D.M., Santini A., Bernatoniene J. (2020). The Essential Oil and Hydrolats from Myristica fragrans Seeds with Magnesium Aluminometasilicate as Excipient: Antioxidant, Antibacterial, and Anti-inflammatory Activity. Foods.

[66] Han Z., Gao J., Wang X., Wang W., Dong J., Zhang Y., Wang S. (2019). Formation and Alterations of the Potentially Harmful Maillard Reaction Products during the Production and Storage of Brown Fermented Milk. Molecules.

[67] Pimenta A.S., Fasciotti M., Monteiro T.V.C., Lima K.M.G. (2018). Chemical Composition of Pyroligneous Acid Obtained from Eucalyptus GG100 Clone. Molecules.

[68] Mikulec A., Kowalski S., Sabat R., Skoczylas L., Tabaszewska M., Wywrocka-Gurgu A. (2019). Hemp flour as a valuable component for enriching physicochemical andantioxidant properties of wheat bread. LWT-Food Sci. Technol..

[69] Srivastava R., Bousquières J., Cepeda-Vázquez M., Roux S., Bonazzi C., Rega B. (2018). Kinetic study of furan and furfural generation during baking of cake models. Food Chem..

[70] Qi L., Zhang K., Wang Y.-T., Wu J.-K., Sui Y., Liang X.-Z., Yu L.-Z., Wu X.-C., Wang P.-M., Xu J.-Z. (2019). Global analysis of furfural induced genomic instability using a yeast model. Appl. Environ. Microbiol..

[71] Ávila M., Garde S., Fernández-García E., Medina M., Nuñez M. (2006). Effect of High-Pressure Treatment and a Bacteriocin-Producing Lactic Culture on the Odor and Aroma of Hispánico Cheese: Correlation of Volatile Compounds and Sensory Analysis. J. Agric. Food Chem..

[72] Song C., Härtl K., McGraphery K., Hoffmann T., Schwab W. (2018). Attractive but toxic: Emerging roles of glycosidically bound volatiles and glycosyltransferases involved in their formation. Mol. Plant.

