# Supplementary material for: Shift of Volatile Organic Compounds (VOCs) in Gluten-Free Hemp-Enriched Sourdough Bread: A Metabolomic Approach
Source: Nutrients. 2020 Apr 10;12(4):1050. doi: 10.3390/nu12041050 (PMC7230689; doi:10.3390/nu12041050)
Supplement: Supplementary file 1 [file nutrients-12-01050-s001.pdf]

Table S1. Proximate composition of flours (w/w).

Flour types	Protein (%)	Carbohydrates (%)	Fiber (%)	Fats (%)	Energy (kJ/100g)
**Maize[1]**	8.5	76.1	2.1	1.3	1503
**Rice[2]**	7.0	79.0	1.0	0.5	1489
**Hemp[3]**	31.0	3.1	49.0	8.1	1253

Commercial organic flours from: [1]maize (Molino Rossetto SpA, Pontelongo, Italy), [2]rice (Molino Rossetto SpA, Italy), [3]hemp seed (Hanf & Natur, Lindlar, Germany).

Table S2. Dough formulations.

Dough types	Maize[1] (g)	Rice[2] (g)	Hemp[3] (g)	Sourdough (g)	NaCl (g)	sterile water (ml)	HPMC[4] (g)
**H**	302.32	75.20	21.27		4.00	340.00	5.60
**YH+**	241.86	60.16	17.73	140.00[5]	3.20	272.00	4.48
**S**	319.40	80.96			4.00	340.00	5.60
**YS+**	255.52	64.77		140.00[6]	3.20	272.00	4.48

Commercial organic flours from: [1]maize (Molino Rossetto SpA, Pontelongo, Italy), [2]rice (Molino Rossetto SpA, Italy), [3]hemp seed (Hanf & Natur, Lindlar, Germany); [4]HPMC = Hydroxy propyl methyl cellulose (Bioline Integratori, Canaro, Italy); [5]LAB fermented hemp seed enriched dough; [6]LAB fermented maize/rice doug.

Table S3. pH values.

Sample	Baseline			End of fermentation		
**SX**	**5.95**	**±**	**0.02^{c}**	4.70	±	0.08^{b}
SL	5.50	±	0.02^{b}	3.60	±	0.08^{a}
SY	5.80	±	0.03^{c}	4.92	±	0.15^{b}
HX	5.94	±	0.04^{c}	5.35	±	0.17^{b}
HL	6.27	±	0.04^{c}	4.14	±	0.12^{a}
HY	6.36	±	0.03^{c}	4.40	±	0.21^{b}
YS	5.80	±	0.03^{c}	5.92	±	0.06^{c}
YS+	5.09	±	0.09^{b}	4.22	±	0.07^{a}
YH	6.36	±	0.01^{c}	5.81	±	0.11^{c}
YH+	4.91	±	0.12^{b}	4.11	±	0.12^{a}

Different letters indicate statistical significance (at least $p<0.05$). For samples abbreviations see Table 1.

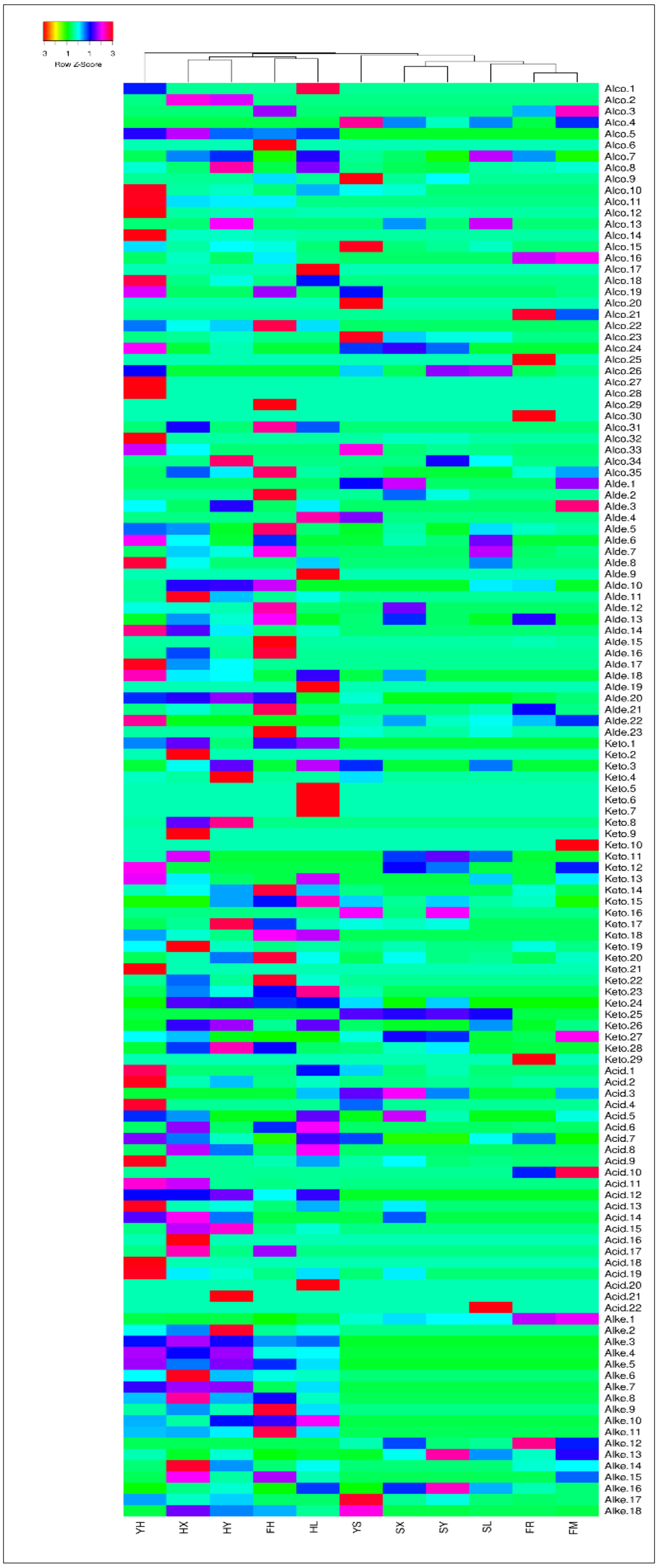

Figure S1. Heatmap of relative quantification of means of VOCs of flour samples and not fermented dough samples. Alco 1 = Propanol methyl; Alco 2 = Butanol, methyl acetate; Alco 3 = Butanetriol; Alco 4 = 1-Pentanol; Alco 5 = Thymol; Alco 6 = 2-Furanmethanol; Alco 7 = 1- Hexanol; Alco 8 = 1-hexanol 2 ethyl; Alco 9 = Hexanol trimethyl; Alco 10 = 1-Heptanol; Alco 11 = Hepten-1-ol (E); Alco 12 = 1-Octanol; Alco 13 = Octenol; Alco 14 = 2-octen-1-ol, (E); Alco 15 = 1-Octanol, 2-butyl; Alco 16 = 3,5 Octadien-2-ol; Alco 17 = 1-Nonanol; Alco 18 = 1-Nonen-3-ol; Alco 19 = 2-Decanol; Alco 20 = 2-Decen-1-ol (E); Alco 21 = Decadienol (E,E); Alco 22 = Borneol; Alco 23 = 2-Undecanol; Alco 24 = 2-Dodecanol; Alco 25 = Isotridecanol; Alco 26 = 2-Tetradecanol; Alco 27 = 1-Hexadecanol; Alco 28 = 2-Heptadecanol; Alco 29 = 1,2 Cyclopentanediol 3 mehtyl; Alco 30 = Cyclohexanol; Alco 31 = Terpineol; Alco 32 = Benzyl

Alcohol; Alco 33 = Ethanol, 2,2'-oxybis; Alco 34 = Phenol; Alco 35 = Phenylethyl alcohol; Alde 1 = Acetaldehyde; Alde 2 = Butanal 3 methyl; Alde 3 = 2 Butenal 2 methyl (E); Alde 4 = Furfural; Alde 5 = Hexanal; Alde 6 = Hexenal, (E); Alde 7 = Heptanal; Alde 8 = 2-Heptenal (Z); Alde 9 = 2,4-Heptadienal (E,E); Alde 10 = Octanal; Alde 11 = 2 Octenal 2 butyl; Alde 12 n= Nonanal; Alde 13 = 2 Nonenal (Z); Alde 14 = 2,4-Nonadienal, (E,E); Alde 15 = Nonadienal; Alde 16 = 2-Decenal (E); Alde 17 = 2,4-Decadienal; Alde 18 = Dodecanal; Alde 19 = Tetradecanal; Alde 20 = 7 Hexadecenal (Z); Alde 21 = Octadecenal; Alde 22 = Benzaldehyde; Alde 23 = Benzeneacetaldehyde; Keto 1 = Ethanone; Keto 2 = Propanone cyclopropyl; Keto 3 = Butanone; Keto 4 = Butanedione; Keto 5 = Pentanone; Keto 6 = 1-Pentanone-2-hydroxy; Keto 7 = Pentanedione; Keto 8 = Ethylcyclopentanone; Keto 9 = Cyclopentenone hydroxy; Keto 10 = Hexanone; Keto 11= Hexenone; Keto 12= Hexanone methyl; Keto 13 = Cyclohexanone butenyl; Keto 14= Heptanone; Keto 15 = Heptenone methtyl; Keto 16 = Heptanone dimethyl; Keto 17 = Octanone; Keto 18 = Octenone; Keto 19 = Octanone dimethyl; Keto 20 = Octadienone (E,E); Keto 21 = Nonanone; Keto 22 = Trans nonenone; Keto 23 = Decanone; Keto 24 = (R) Bornane dione; Keto 25 = Undecanone; Keto 26 = Undecenone butyl dimethyl (E); Keto 27 = Undecadienone dimethyl (E); Keto 28 n= Gamma dodecalactone; Keto 29 = Pentadecanone; Acid 1 = Ethyl acetate; Acid 2 = Formic acid; Acid 3 = Propanoic acid; Acid 4 = Propanoic acid hydroxy; Acid 5 = 2-Propenoic acid; Acid 6 = Butanoic acid; Acid 7 = Pentanoic acid; Acid 8 = Valeric acid; Acid 9= Hexanoic acid; Acid 10 = Sorbic acid; Acid 11 = Heptanoic acid; Acid 12 = Heptenoic acid; Acid 13 = Octanoic acid; Acid 14 = Nonanoic acid; Acid 15 = Nonenoic acid; Acid 16 = n-Decanoic acid; Acid 17 = Tetradecanoic acid hydroxy; Acid 18 = Pentadecanoic acid; Acid 19 = n-Hexadecanoic acid; Acid 20 = Heptadecanoic acid; Acid 21 = Nonadecanoic acid; Acid 22 = Thiophene acetic acid; Alke 1= Limonene; Alke 2 = Beta-phellandrene; Alke 3 = p-menthatriene; Alke 4 = Delta-3-carene; Alke 5 = Alpha-bergamotene; Alke 6 = Alpha-farnesene; Alke 7 = Beta-caryophillene; Alke 8 = Caryophillene oxyde; Alke 9 = (R) alpha pinene; Alke 10 = Camphene; Alke 11 = Hexadecene (Z) ; Alke 12= Heptadecene; Alke 13 = Octadecene (E); Alke 14 = Nonadecene; Alke 15 = Decene; Alke 16 = Undecene methyl (E); Alke 17 = Tetradecene (E); Alke 18 = Eicosene (E).

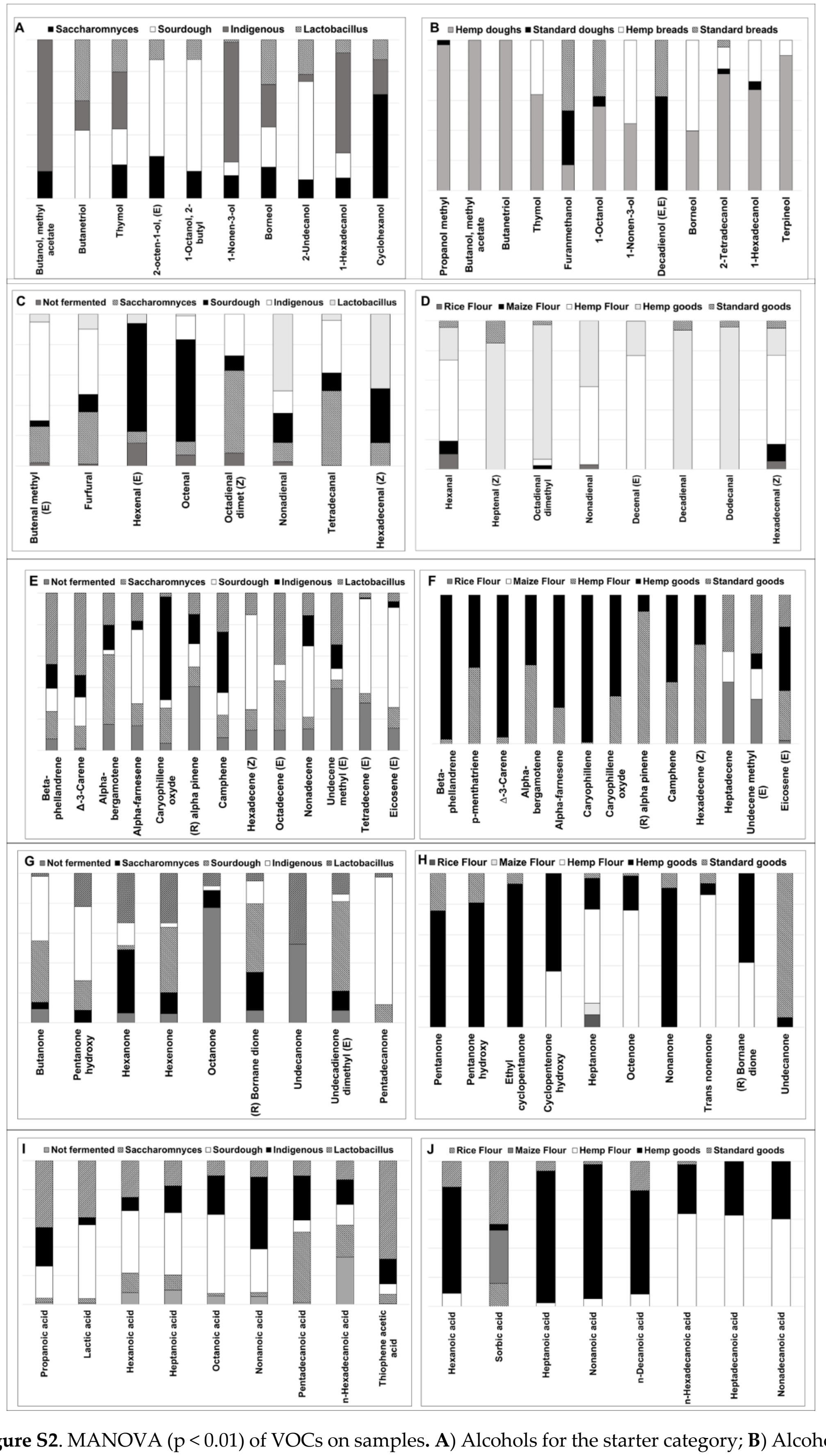

Figure S2. MANOVA ($p < 0.01$) of VOCs on samples**. A**) Alcohols for the starter category; **B**) Alcohols for the matrix category; **C**) Aldehydes for the starter category; **D**) Aldehydes for the matrix category; **E**) Alkenes for the starter category; **F**) Alkenes for the matrix category; **G**) Ketones for the starter category; **H**) Ketones for the matrix category; **I**) Organic acids for the starter category; **J**) Organic acids for the matrix category.
